# Platelet counts are associated with arterial stiffness in Chinese Han population: a longitudinal study

**DOI:** 10.1186/s12872-020-01634-7

**Published:** 2020-07-30

**Authors:** Kuo Liu, Junfeng Xu, Lixin Tao, Kun Yang, Yang Sun, Xiuhua Guo

**Affiliations:** 1 Department of Epidemiology and Health statistics, School of Public Health, Capital Medical University, No.10 Xitoutiao, Youanmenwai Avenue, Beijing, 100069 People’s Republic of China; 2 Beijing Municipal Key Laboratory of Clinical Epidemiology, Beijing, China; 3 Beijing Xiaotangshan Hospital, Xiaotangshan Town, Changping District, Beijing, 102211 China

**Keywords:** Arterial stiffness, Blood pressure, Platelet counts, Longitudinal study

## Abstract

**Background:**

Determining the risk factors for brachial-ankle pulse wave velocity (baPWV) may help to identify people susceptible to diabetic atherosclerosis and could prevent diabetic macrovascular complications in the early stages. We aim to comprehensively investigate risk factors contributing to arterial stiffness in patients with and without diabetes.

**Methods:**

BaPWV was measured in 5651 individuals who attended health check-ups at baseline and follow-up. Lasso regression was used to screen for risk factors. Mixed models and multiple linear regressions were subsequently established to evaluate the effect size of the potential risk factors on baPWV and PWV change rates. All analyses were stratified by diabetes. Mediation analysis was also conducted to demonstrate the mechanisms of arterial stiffness in patients with diabetes.

**Results:**

In lasso regression, postprandial 2-h glucose (P2hG), systolic blood pressure (SBP) and age were associated with baPWV regardless of diabetes. Platelet counts (PLT), mean corpuscular volume (MCV) and coronary heart disease (CHD) were associated with baPWV in patients with diabetes. In the mixed models, PLT were positively associated with baPWV in patients with diabetes (*β*_platelet, perSD_ = 25.80; 18.26–33.33). Elevated PLTs could also significantly increase the PWV change rate in patients with diabetes (*β*_platelet, perSD_ = 54.05; 10.00–107.10). In mediation analysis, diabetes had a significant average direct effect on baPWV. The average causal mediation effect (ACME) of PLTs was 1.76, with a range of 0.17 to 3.70.

**Conclusions:**

Elevated PLT counts can increase baPWV in diabetes and are a potential mediator between diabetes and atherosclerosis.

## Background

Arterial stiffness is an important risk factor for cardiovascular disease (CVD) and is a key element in the pathogenesis of CVD [1]. Growing evidence shows that increased arterial stiffness can increase both cardiovascular mortality and all-cause mortality [2, 3]. The most common noninvasive index used to quantify these estimates of arterial stiffness is brachial-ankle pulse wave velocity (baPWV) [4]. BaPWV can reflect both central and peripheral arterial stiffness [5, 6]. An increase in baPWV has been related to the severity of peripheral vascular disease and higher cardiovascular risk [7].

Studies have demonstrated various risk factors for arterial stiffness. Elevated blood pressure (BP) [8] and long-term systolic blood pressure (SBP) variability [9] were shown to increase arterial stiffness. Type 2 diabetes (T2DM) has also been confirmed to increase arterial stiffness in several large cohort studies [10, 11] and has been found to have causal association with arterial stiffness in a Chinese population [12]. However, the mechanisms of arterial stiffening in T2DM patients are still not clear. Moreover, the risk factors for arterial stiffness may be different between patients with diabetes and patients without diabetes. Misako Hamamura et al. [13] demonstrated several risk factors for arterial stiffness in T2DM patients, including age, systolic blood pressure, serum uric acid (UA), urinary albumin excretion and lower body mass index (BMI). A cross-sectional study carried out by Bo Youn Won et al. [14] showed that age, mean arterial pressure (MAP), pulse rate, waist circumference and gamma-glutamyl transferase (GGT) could increase baPWV in the elderly population without hypertension, diabetes and statin use. Finding the difference in arterial stiffness risk profiles between patients with and without diabetes would be important for both the etiological study of diabetic macrovascular complications and the prevention of arterial stiffness. However, the difference in arterial stiffness risk profiles between non-T2DM and T2DM patients is still not clear and needs to be further studied. Moreover, whether serum lipids, routine blood indexes or biochemical indexes of liver and kidney function contribute to arterial stiffening has seldom been discussed in previous studies. Determining the risk factors related to baPWV in patients with diabetes may help to identify people susceptible to diabetic atherosclerosis and will aid in the prevention of diabetic macrovascular complications in its early stage.

In a longitudinal study using data from health check-ups in China, we attempted to determine whether traditional risk factors together with routine blood indexes and biochemical indexes contributed to the development of arterial stiffness. Lasso regression was used to screen risk factors, and a mixed model and multiple linear regressions were used to evaluate the effect size of potential risk factors. All analyses were stratified by diabetes, and mediation analysis was also conducted to explore the mechanisms of arterial stiffness in patients with diabetes.

## Methods

### Study participants

This longitudinal cohort participated in routine health check-ups in Beijing Xiaotangshan Hospital from 2007 to 2015. All participants had check-ups once a year, and those participants who had at least two check-ups with baPWV measurements were included in the current study. The baPWV and other physical indicators were measured at baseline and each check-up. This study was approved by the Ethics Committee of Capital Medical University (No: 2016SY24). Anthropometric and laboratory measurements were performed at each health check-up, and all participants provided consent.

### Measurements

The participants’ medical history of chronic disease and medication history were collected by physicians during routine physical examinations. Information on lifestyle risk factors (cigarette smoking, alcohol consumption, etc.) was collected from participants through a face-to-face interview by trained interviewers using uniform questionnaires.

A mercury sphygmomanometer was used to measure blood pressure. Blood pressure measurements were obtained at least 30 min after participants had smoked or had caffeinated products. Blood pressure was measured three times at the right brachial artery while the participant was in a seated position. The average of the last two measurements was used for data analysis.

The baPWV was measured using an automatic waveform analyzer (BP-203RPE II; OMRON, Japan). Participants were asked to rest 5 min in the supine position before measurements were performed. Occlusion and monitoring cuffs were wrapped around the subjects’ arms and ankles. Pulse volume waveforms of the bilateral brachial and posterior tibial arteries were recorded simultaneously to determine the time interval between brachial and tibial waveforms. The height of the participants was used to calculate the transmission distance from the brachium to the ankle. The mean of the right-side and left-side baPWV values was used for analysis. Measurements were conducted by well-trained doctors who were blinded to the clinical information. All physical examinations were performed in the morning after fasting.

After overnight fasting, venous blood samples were collected from all participants for fasting blood analysis and again for an OGTT. Venous blood samples were obtained and stored in a 4 °C refrigerator. All hematological analyses were performed within 8 h. Serum glucose and biochemical parameters were measured by an enzymatic method using a chemistry analyzer (Beckman LX20, Beckman, Brea, CA, USA) in the central laboratory of the hospital. Platelet counts in blood samples collected in EDTA tubes were determined based on the Coulter principle using an automated differential cell counter (Beckman Coulter Act5Diff, Beckman Coulter®).

### Definition of atherosclerotic risk factors

Smoking status was determined through three alternatives: “current smoking”, “past smoking”, or “rare/never smoking”. Current smoking was defined as at least 1 cigarette per day over a period of more than 1 year. Not smoking for at least 3 months was defined as past smoking. Alcohol consumption was determined by three alternatives: “current drinking”, “past drinking”, or “rare/never drinking”. Current drinking was defined as drinking at least once per week for the previous month. Drinking at least once per week but not within the last month was defined as past drinking. Rare/never drinking was defined as never drinking or drinking less than once a week.

T2DM was defined according to the 1999 World Health Organization diagnostic criteria as fasting plasma glucose (FPG) ≥7.0 mmol/L or self-reported physician-diagnosed diabetes and/or taking antidiabetic medication. Hypertension was defined as systolic blood pressure ≥ 140 mmHg and/or diastolic blood pressure ≥ 90 mmHg and/or currently taking antihypertensive medication. Coronary heart disease (CHD) was defined as nonfatal ischemic heart disease, including acute myocardial infarction and angina pectoris.

### Statistical analysis

SAS (version 9.4; SAS Institute, Chicago, IL, USA) and R (version 3.6.0) were used for database management and statistical analysis. Continuous variables with normal distribution are expressed as the means ± standard deviations (SDs), while continuous variables without normal distribution are expressed as medians ± interquartile range. Categorical variables are expressed as percentages (%). To compare the difference in each continuous variable between patients with diabetes and patients without diabetes, Student’s *t* test or Wilcoxon rank sum test were used.

Lasso regressions were performed for the participants with questionnaires to screen risk factors for baPWV using the R software glmnet package. The included factors were demographic characteristics: age and sex; lifestyle risk factors: smoking and drinking; disease history: coronary heart disease, hypertension, and type 2 diabetes; medications: antidiabetic drugs, antihypertensive drugs, and lipid-lowering drugs; anthropometric measurements; systolic blood pressure (SBP); diastolic blood pressure (DBP); body mass index (BMI); blood lipid measurements: high density lipoprotein cholesterol (HDL-C), low density lipoprotein cholesterol (LDL-C), total cholesterol (TC), and triglyceride (TG) levels; blood glucose measurements: fasting plasma glucose (FPG) and postprandial 2-h glucose (P2hG); routine blood measurements: red blood cell count (RBC), mean corpuscular volume (MCV), mean corpuscular hemoglobin (MCH), mean corpuscular hemoglobin concentration (MCHC), red cell distribution width (RDW), white blood cell (WBC) count, lymphocyte (LYM) count, lymphocyte percentage (LYM%), monocyte (MONO) count, monocyte percentage (MONO%), eosinophil (EO) count, eosinophil percentage (EO%), basophil (BASO) count, basophil percentage (BASO%), neutrophil (NEUT) count, percentage of neutrophil (NEUT%), hemoglobin (HGB), platelet (PLT) counts, mean platelet volume (MPV), platelet volume distribution width (PDW), plateletcrit (PCT), and erythrocyte sedimentation rate (ESR); and blood biochemical indexes including ① liver function indicators: total bilirubin (TBIL), glutamyl transpeptidase (GGT), albumin (ALB), globulin (GLB), albumin-to-globulin ratio (AG), alanine aminotransferase (ALT), and aspartate aminotransferase (AST) and ② renal function indicators: creatinine (CR), urea nitrogen (BUN), and serum uric acid (UA).

When lasso regressions were conducted for the DM group and non-DM group, “DM” was excluded from the variables mentioned above. Linear mixed-effects models and multiple linear regressions were subsequently conducted in all participants using the SAS mixed procedure to evaluate the effect size of potential risk factors determined by lasso regressions.

Mediation analysis to uncover causal pathways linking diabetes to baPWV was performed by R software mediation packages. The main outputs from the mediation function were total effect (TE), average causal mediation effect (ACME), and average direct effect (ADE). A two-sided *P* < 0.05 was considered statistically significant.

## Results

A total of 5651 participants underwent baPWV measurement in the longitudinal study with a mean follow-up of 5.0 years. The mean age of the participants was 58.0. The age of patients without diabetes (66.80 ± 12.11) was significantly higher than that of patients with diabetes (56.97 ± 13.56). BMI and SBP were significantly higher in patients without diabetes, while DBP was significantly higher in patients with diabetes. The percentage of males was significantly higher in patients with diabetes (87.9% vs. 73.5%, *P* < 0.001). Most of the routine blood indexes and biochemical indexes, except for TG, UA, MCH, MPV, TP, TBIL, ALT and PLT, were significantly different between patients with and without diabetes. The proportions of patients who reported smoking and drinking were not significantly different between the patients with DM and the patients without DM. Details are shown in Table 1.

**Table 1:** Baseline characteristics of the study participants

	All	DM	non-DM	*P* value
Age (year)	58.00 ± 13.74	56.97 ± 13.56	66.80 ± 12.11	< 0.001**
Male^b^	4240 (75.0)	517 (87.9)	3723 (73.5)	< 0.001**
Current Smoking^b^	232 (30.4)	14 (20.6)	218 (31.3)	0.073
Current Drinking^b^	268 (35.1)	18 (36.0)	250 (35.0)	0.145
Body mass index (kg/m^2^)	25.27 ± 4.76	25.20 ± 4.74	25.82 ± 4.90	0.003*
Systolic blood pressure (mmHg)	123.82 ± 16.31	123.15 ± 16.28	129.46 ± 15.45	< 0.001**
Diastolic blood pressure (mmHg)	78.10 ± 9.39	78.25 ± 9.45	76.84 ± 8.78	0.001*
Mean arterial pressure (mmHg)	93.34 ± 10.61	93.22 ± 10.74	94.38 ± 9.38	0.006*
High density lipoprotein cholesterol^a^(mmol/L)	1.29 ± 0.42	1.18 ± 0.38	1.30 ± 0.42	< 0.001**
Low density lipoprotein cholesterol (mmol/L)	3.12 ± 0.83	3.13 ± 0.82	3.02 ± 0.84	0.002*
Triglyceride^a^(mmol/L)	1.40 ± 1.03	1.38 ± 1.11	1.40 ± 1.03	0.729
Total cholesterol (mmol/L)	4.93 ± 0.94	4.74 ± 0.99	4.95 ± 0.93	< 0.001**
Hypertension^b^	2515 (44.5)	375 (63.8)	2140 (42.3)	< 0.001**
Coronary heart disease^b^	467 (8.3)	127 (21.5)	340 (6.7)	< 0.001**
Brachial-ankle pulse wave velocity (cm/s)	1407.25 ± 374.75	1683.22 ± 317.96	1451.40 ± 291.94	< 0.001**
**Blood routine measurements**				
Red blood cell count (RBC)	4.65 ± 0.45	4.62 ± 0.46	4.66 ± 0.44	0.058
Red cell distribution width (%)	11.92 ± 1.28	11.99 ± 1.42	11.91 ± 1.26	0.120
Hemoglobin (g/L)	143.48 ± 14.72	143.62 ± 14.76	142.33 ± 14.29	0.045*
Mean corpuscular hemoglobin (pg)	30.96 ± 1.96	30.95 ± 1.96	31.0 ± 1.88	0.562
Mean corpuscular hemoglobin concentration (g/L)	338.33 ± 13.67	338.46 ± 13.65	337.28 ± 13.74	0.050
Mean Corpuscular Volume (fL)	91.40 ± 5.26	91.90 ± 4.91	91.33 ± 5.28	0.012*
Mean platelet volume (fL)	8.46 ± 1.08	8.46 ± 1.06	8.45 ± 1.17	0.883
Platelet volume distribution width (fL)^a^	8.70 ± 4.20	9.89 ± 4.10	8.70 ± 4.20	0.048*
Platelet crit (%)	0.17 ± 0.04	0.16 ± 0.04	0.17 ± 0.04	< 0.001**
Platelet counts (10^9^/L)	192.91 ± 48.47	177.79 ± 44.93	194.70 ± 48.57	< 0.001**
White blood cell count (WBC)	5.86 ± 1.47	5.92 ± 1.46	5.85 ± 1.47	0.309
Monocyte percentage (%)	3.66 ± 2.65	4.05 ± 4.55	3.61 ± 2.35	0.302
Monocyte count (10^9^/L)	0.29 ± 0.16	0.31 ± 0.27	0.29 ± 0.15	0.200
Neutrophil percentage (%)	58.75 ± 7.51	60.04 ± 7.30	58.60 ± 7.51	< 0.001**
Neutrophil count (10^9^/L)	3.45 ± 1.11	3.54 ± 1.12	3.44 ± 1.11	0.105
Percentage of lympholeukocyte (%)	35.70 ± 7.27	35.79 ± 7.30	34.94 ± 6.98	0.008*
Lympholeukocyte (10^9^/L)	2.04 ± 0.55	2.04 ± 0.56	2.02 ± 0.53	0.027*
Eosinophils percentage (%)	3.10 ± 2.02	3.21 ± 1.88	3.09 ± 2.03	0.422
Eosinophils count (10^9^/L)	0.19 ± 0.19	0.20 ± 0.13	0.19 ± 0.20	0.514
Basophils percentage (%)	0.38 ± 0.22	0.35 ± 0.20	0.39 ± 0.23	0.011
Basophils count (10^9^/L)	0.02 ± 0.01	0.02 ± 0.01	0.02 ± 0.01	0.171
Erythrocyte sedimentation rate (mm/h)^a^	6.40 ± 8.20	6.30 ± 8.00	7.40 ± 8.90	< 0.001**
**Glucose**				
Fasting plasma glucose (mmol/L)^a^	5.33 ± 0.85	6.76 ± 2.00	5.26 ± 0.74	< 0.001**
Postprandial 2 h-glucose (mmol/L)^a^	6.50 ± 2.20	9.30 ± 4.00	6.30 ± 2.00	< 0.001**
**Blood biochemical indexes**				
Total protein (g/L)	71.01 ± 4.28	71.02 ± 4.28	70.92 ± 4.22	0.586
Albumin (g/L)	44.30 ± 2.702	44.33 ± 2.70	44.09 ± 2.69	0.040*
Globulin (g/L)	27.11 ± 3.59	27.23 ± 3.66	27.10 ± 3.59	0.405
Albumin and globulin ratio	1.67 ± 0.25	1.67 ± 0.25	1.65 ± 0.26	0.110
Total bilirubin (μmol/L)	15.65 ± 5.78	15.65 ± 5.82	15.66 ± 5.41	0.960
Gamma-glutamyl transferase (U/L)^a^	23.00 ± 19.00	21.00 ± 15.00	23.00 ± 19.00	0.002*
Alanine aminotransferase (U/L)^a^	21.00 ± 14.00	20.00 ± 12.00	21.00 ± 14.00	0.155
Aspartate aminotransferase (U/L)	20.00 ± 7.00	20.00 ± 7.00	21.00 ± 6.00	0.001*
Creatinine (μmol/L)	90.11 ± 19.89	89.58 ± 19.86	94.60 ± 19.68	< 0.001**
Uric acid (mmol/L)	346.56 ± 88.10	346.02 ± 88.50	351.15 ± 84.59	0.183
Blood urea nitrogen (mmol/L)	5.59 ± 1.47	6.33 ± 1.68	5.50 ± 1.42	< 0.001**

^a^Continuous variables without normal distribution are expressed as medians ± interquartile range

^b^Categorical variables were presented as number (percentage)

* *P* < 0.05; **P < 0.001

Lasso regression was used to screen for risk factors for baPWV. P2hG, age, and SBP were associated with follow-up baPWV in the whole population, patients with diabetes, and patients without diabetes. Hypertension was associated with follow-up baPWV in the whole population and in patients with diabetes. PLT, MCV and CHD were associated with follow-up baPWV only in patients with diabetes.

Mixed models were subsequently used to determine the effect size of potential risk factors selected by lasso regression. All risk factors associated with baPWV in lasso regression were included in the mixed models. Risk factors associated with baPWV are listed in Table 2. P2hG, age and SBP were significantly associated with baPWV regardless of whether the results were stratified by diabetes (*P* < 0.001). Hypertension and higher PLT can significantly increase baPWV in patients with diabetes. The interaction between DM and PLT was statistically significant in all participants (*P* = 0.0016).

**Table 2:** Risk factors associated with baPWV in mixed models

	All		DM		Non-DM	
	beta (95%CI)	*P* value	beta (95%CI)	*P* value	beta (95%CI)	*P* value
Age (year)	12.69 (12.44,12.92)	< 0.001*	12.47 (12.20,12.74)	< 0.001*	14.64 (13.78,15.48)	< 0.001*
Postprandial 2 h-glucose (mmol/L)	10.46 (9.24,11.68)	< 0.001*	10.38 (8.71,12.05)	< 0.001*	6.42 (3.81,9.04)	< 0.001*
Systolic blood pressure (mmHg)	5.95 (5.93,6.18)	< 0.001*	5.98 (5.74,6.21)	< 0.001*	5.76 (5.14,6.38)	< 0.001*
Hypertension	NA	NA	25.80 (18.26,33.33)	< 0.001*	NA	NA
Platelet counts (10^9^/L, per SD)	NA	NA	10.77 (5.91,20.63)	< 0.001*	NA	NA
Mean corpuscular volume (fL)	NA	NA	0.04(−0.53,0.61)	0.891	NA	NA
Coronary heart disease	NA	NA	−2.48(−14.03,9.07)	0.673	NA	NA
Interaction term: DM × PLT^a^	0.177 (0.067, 0.287)	0.0016*	NA	NA	NA	NA

Only variables which were significantly associated with baPWV in lasso regression were included in mixed models

^a^In all participants, interaction terms of diabetes (DM) and platelet counts (PLT) were added in mixed models, adjusting for age, Postprandial 2 h-glucose, Systolic blood pressure

*P value is significant after Bofferoni correction

**Fig. 1:**
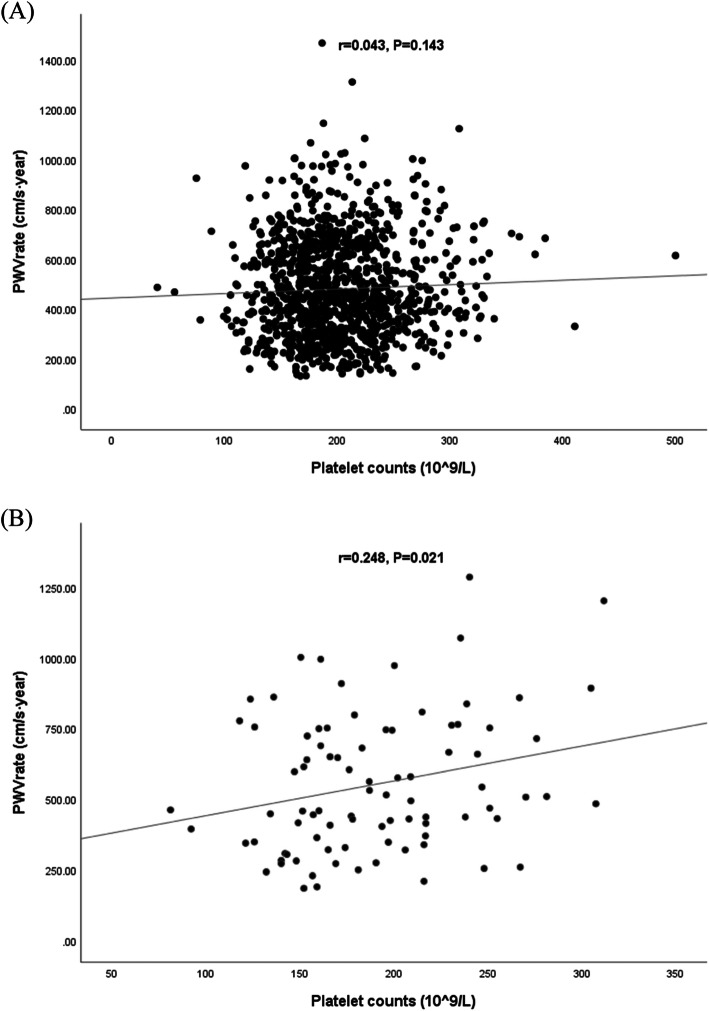
Scatter plot of measurements and baPWV change per year stratified by diabetes. **a** Scatter plot of platelets counts and PWV change rate per year in non-diabetes. **b** Scatter plot of platelets counts and PWV change rate per year in diabetes

Multiple linear regressions were also used to explore the effect size of risk factors on the PWV change rate. All risk factors associated with baPWV in lasso regression were included in the regression models. Elevated PLT and P2hG increased the PWV change rate in diabetes (*β*_*PLT, per SD*_ = 54.05, 10.00–107.10, *P*_*PLT*_ = 0.046; *β*_P2hG_ = 13.85, 2.50–25.21, *P*_P2hG_ = 0.017, respectively). Age, P2hG and SBP were significantly associated with the baPWV change rate in both the whole population and patients without diabetes. Details are shown in Table 3. The linear relationship between PLT and baPWV change rate per year in patients with diabetes is shown in Fig. 1.

**Table 3:** Risk factors associated with PWV change rate in multiple linear regressions

	all		DM		Non-DM	
	beta (95%CI)	*P* value	beta (95%CI)	*P* value	beta (95%CI)	*P* value
Age (year)	2.562 (1.538,3.585)	< 0.001**	0.511(−4.278, 5.300)	0.832	2.900 (1.841,3.958)	< 0.001*
Postprandial 2 h-glucose (mmol/L)	10.139 (6.041,14.236)	< 0.001**	13.851 (2.497,25.205)	0.017*	8.005 (2.983,13.028)	0.002*
Systolic blood pressure (mmHg)	1.793 (0.792,2.794)	< 0.001**	3.254(−1.567,8.076)	0.183	1.570 (0.750,2.390)	< 0.001**
Hypertension	−4.511(−35.319,26.297)	0.774	26.461(− 115.621,168.543)	0.712	NA	NA
Platelet counts (10^9^/L, per SD)	NA	NA	54.05 (10.00, 107.10)	0.046*	NA	NA
Mean Corpuscular Volume (fL)	NA	NA	8.967(−2.993,20.927)	0.139	NA	NA
Coronary heart disease	NA	NA	−75.009(− 222.022,72.004)	0.313	NA	NA

Only variables which were significantly associated with baPWV in lasso regression were included in mixed models

* *P* < 0.05; **P < 0.001

Anthropometry and biomarkers significantly associated with baPWV in the diabetes group were subsequently examined for their mediation effects between diabetes and baPWV. The results of the mediation analysis are shown in Table 4. Diabetes had a significant average direct effect (ADE) on baPWV in each model (*P* < 0.05). The average causal mediation effect (ACME) of DBP is 3.219, with a range of 1.327 to 5.860 (*P* < 0.001). PLT also had significant ACME with a range of 0.17 to 3.70 (ACME = 1.761, *P* = 0.021).

**Table 4:** Mediation analysis between diabetes and baPWV progression rate

		ACME	ADE	Total Effect	Prop. Mediated
Systolic blood pressure (mmHg)	Estimate(95%CI)	0.012 (0.004, 0.436)	23.92 (4.09, 44.37)	23.93 (4.23, 44.44)	4.18 × 10^−4^ (3.15 × 10^−6^, 2.03 × 10^− 2^)
	*P* value	0.972	0.020*	0.021*	0.963
Diastolic blood pressure (mmHg)	Estimate(95%CI)	3.219 (1.327,5.860)	21.10 (1.60,40.73)	24.31 (4.28,44.23)	0.130 (0.041,0.600)
	*P* value	< 0.001**	0.048*	0.012*	0.012*
Platelet counts (10^9^/L)	Estimate(95%CI)	1.761 (0.174,3.702)	22.08 (3.49,40.85)	23.83 (5.45,43.50)	0.071 (0.008,0.332)
	*P* value	0.021*	< 0.001**	< 0.001**	0.021*

* *P* < 0.05; **P < 0.001

## Discussion

In the current longitudinal study, we found that higher blood pressure can increase the risk of arterial stiffness in both patients with diabetes and patients without diabetes. DBP and PLT may be causally associated with baPWV in patients with diabetes. The most important strengths of the current longitudinal study were a relatively large sample size with a median follow-up of 5 years. Moreover, traditional risk factors together with routine blood indexes and biochemical indexes were included to screen for potential risk factors for baPWV.

It has long been believed that BP is related to arterial stiffness because elastic arterial walls will change in response to chronically elevated blood pressures [15]. The relationship between DBP and arterial stiffness is not consistent. In the Georgia Cardiovascular Twin Study, DBP showed strong positive associations with PWV in American youth and young adults [16]. In a population with a mean age of 68, both systolic hypertension alone and combined with diastolic hypertension were found to be significantly associated with PWV [17]. However, Franklin SS et al. found that a fall in DBP after age 60 was associated with a continual rise in SBP, which was consistent with increased large artery stiffness [18]. Blood pressure (BP) will fluctuate from moment to moment depending on patient- and observer-related factors; thus, the measurement of BP is complicated. In the current study, we did not retake BP measurements for the participants who had abnormal BP. Thus, it was likely that elevated DBP found in a routine health examination was only a transient abnormality. Wingfield D et al. [19] demonstrated that transient high DBP could increase both cardiovascular (CVD) mortality and the incidence of diabetes. Given that arterial stiffness is independently predictive of CVD death [20], transient high DBP may also be associated with arterial stiffness. Moreover, in the current study, we found that DBP is a significant mediator between DM and arterial stiffness. In insulin-resistant patients, plasma asymmetric dimethylarginine (ADMA), which antagonizes endothelium-dependent vasodilatation, was increased independent of hypertension [21]. Moreover, vasodilation in response to insulin concentrations was reduced in insulin-resistant patients [22]. Endothelium-dependent vasodilation dysfunction was related to higher DBP [23]. Thus, patients with diabetes with insulin resistance may have a higher DBP because of endothelial dysfunction. Brachial DBP is a useful marker of central BP [24], and DBP was previously found to be associated with central arterial stiffness, which was measured by carotid-femoral pulse wave velocity (cfPWV) [25]. Thus, a positive association between DBP and baPWV may indicate a role of endothelial dysfunction in central arterial stiffness.

In this study, we found that elevated PLT can increase baPWV in diabetes, and it may be causally associated with arterial stiffness in diabetes. Type 2 diabetes is characterized by insulin resistance and β-cell dysfunction. Insulin resistance is typically associated with hyperinsulinemia, which results from the feedback response to the loss of the intracellular signaling pathway on glucose transportation. An epidemiological study demonstrated that increased plasminogen activator inhibitor type 1 (PAI-1) levels were associated with type 2 diabetes mellitus and may be a component of insulin-resistance syndrome [26]. Other studies aimed at mimicking the metabolic pattern of type 2 diabetes showed that hyperinsulinemia could cause a sustained increase in the concentration of PAI-1 in the blood in normal subjects [27, 28]. Levels of PAI-1 have been shown to be positively associated with ischemic heart disease, and overexpression of PAI-1 mRNA has been observed in atherosclerotic human arteries, suggesting that PAI-1 is involved in the development and progression of atherosclerosis [29–31]. Several studies have shown that platelet (PLT) counts were significantly correlated with PAI-1 (*r* = 0.44) and platelet aggregation (*r* = 0.11) [32]. Thus, patients with diabetes with hyperinsulinemia experience PAI-1 elevation and platelet aggregation, which are involved in the pathophysiology of atherosclerosis. Given that PLT is positively correlated with PAI-1 and platelet aggregation, the identification of PLT as a mediator in the current study might indicate the underlying mechanisms of diabetic atherosclerosis. Moreover, Robert H. Lee et al. [33] demonstrated another possible mechanism by which platelet numbers contribute to the pathology of diabetic atherosclerosis. Elevated blood glucose levels can trigger S100 calcium-binding protein A8/A9 (S100A8/A9) and could ultimately lead to increased thrombopoietin (TPO) production by binding to a receptor of advanced glycation end-products (RAGE). TPO causes megakaryocyte proliferation and increased platelet production. Dysregulation of the number and function of platelets might contribute to the development of atherosclerosis.

Many endothelium-derived factors involved in the regulation of blood coagulation may be altered in diabetes, leading to initial platelet adhesion and subsequent platelet aggregate formation. The interaction of activated platelets with endothelial cells and leukocytes, together with the coagulation system, may contribute to the process of atherosclerosis [34]. Mean platelet volume (MPV) is a marker of platelet size and activity. Platelets from diabetic patients are characterized by dysregulation of several signaling pathways [35]. Diabetes can enhance the inflammatory response to bacteria both systemically and at the site of infection [36]. Smaller platelets are considered inflammatory markers in many infectious diseases and are associated with chronic inflammatory processes [37, 38]. In addition, inflammation is also important in the pathogenesis of arterial stiffness and atherosclerosis [39, 40]. A population-based study found that MPV is positively associated with vascular stiffness, and larger platelets are generally considered a strong indicator of atherosclerosis [41].. Thus, the effect of MPV on arterial stiffness in patients with diabetes may be a more complex situation compared with that in the general population. However, few studies have focused on the effect of MPV on arterial stiffness in diabetes. The current study found that only platelet counts could increase baPWV in diabetes but did not find a significant association between MPV and the risk of arterial stiffness. Further studies are still needed in the future to explore the role of platelets and their activity on arterial stiffness in patients with diabetes.

There are some limitations of the current study. First, the patients’ history of disease and medication were self-reported. To minimize bias, the above information was acquired by professional physicians. Second, not all participants completed questionnaires, so it was difficult to evaluate the impact of lifestyle risk factors on arterial stiffness in all participants. We performed lasso regression only in the participants with complete questionnaires. Neither smoking nor drinking was found to be a risk factor for arterial stiffness in lasso regression; thus, the following analyses were performed in all participants without considering their lifestyle risk factors. Third, the current study is a physical examination-based cohort instead of a community-based cohort study. Due to the different possibility for people to choose baPWV measurement in their health examination, the current study may have selection bias to a certain extent. Only participants with more than two baPWV measurements were included in the study, which may also induce selection bias. Older patients and those with metabolic cardiovascular disease might preferentially choose baPWV measurement in their health check-ups several times. To cover this shortage, we adjusted for age, coronary heart disease, blood lipids and medication in the lasso regression model. In addition, people who chose to undergo repeated baPWV measurements might have characteristics that are difficult to measure quantitatively, for example, their health literacy or socioeconomic status. Thus, the results need to be further verified in community-based cohorts. In addition, blood indicators included in the current study are routine items for health check-ups. Because HbA1c was not a routine item and participants seldom chose to test for it in their check-ups, the effect of long-term blood glucose control on atherosclerosis could not be fully evaluated. Moreover, in this research, we cannot determine the mechanism behind the mediation analysis from DM patients relating platelet count to PWV. More research on the potential mechanisms by which platelets increase the risk of diabetic atherosclerosis is needed in the future.

## Conclusions

In the current study, we have found that platelet (PLT) count is positively associated with baPWV in a diabetic population, and PLT count may be a mediator linking diabetes to arterial stiffness. Our findings indicate that PLT count has the potential to be a predictor of atherosclerosis in patients with diabetes, and the identification of PLT count as a predictor might contribute to the early prevention of diabetic macrovascular complications.

## Data Availability

The datasets generated during the current study are not publicly available yet. Data can be made available on reasonable request through contacting the corresponding author.

## Abbreviations

LYM: Absolute value of lymphocyte count
ALT: Alanine aminotransferase
ALB: Albumin
AGR: Albumin and globulin ratio
AST: Aspartate aminotransferase
ACME: Average causal mediation effect
ADE: Average direct effect
BASO: Basophils count
BASO%: Basophils percentage
BP: Blood pressure
BMI: Body mass index
BUN: Blood urea nitrogen
baPWV: Brachial-ankle pulse wave velocity
CVD: Cardiovascular disease
CHD: Coronary heart disease
CR: Creatinine
DBP: Diastolic blood pressure
EO: Eosinophils count
EO%: Eosinophils percentage
ESR: Erythrocyte sedimentation rate
FPG: Fasting plasma glucose
GGT: Gamma-glutamyl transferase
GLB: Globulin
GGT: Glutamyl transpeptidase
HGB: Hemoglobin
PLT: Platelet counts
PCT: Platelet crit
HDLC: High density lipoprotein cholesterol
LDLC: Low density lipoprotein cholesterol
MAP: Mean arterial pressure
MCH: Mean corpuscular hemoglobin
MCHC: Mean corpuscular hemoglobin concentration
MCV: Mean corpuscular volume
MPV: Mean platelet volume
MONO: Monocyte count
MONO%: Monocyte percentage
NEUT: Neutrophil count
NEUT%: Neutrophil percentage
LYM%: Lympholeukocyte percentage
PDW: Platelet volume distribution width
P2hG: Postprandial 2 h-glucose
RBC: Red blood cell count
RDW: Red cell distribution width
SDs: Standard deviations
SBP: Systolic blood pressure
TBIL: Total bilirubin
TC: Total cholesterol
TE: Total effect
TP: Total protein
TG: Triglyceride
T2DM: Type 2 diabetes
UA: Uric acid
WBC: White blood cell count
